# The protective effects of active ingredients from acrorus tatarinowii on sperm and their molecular mechanisms

**DOI:** 10.1186/s12610-024-00247-w

**Published:** 2025-01-20

**Authors:** Zonglin Lu, Haiyang Zhao, Hui Wang, Xin Wang, Zixue Sun

**Affiliations:** 1 The Second Clinical Medical College, Henan University of Traditional Chinese Medicine, No. 7, Jiudu Road, Xigong District, Zhengzhou, Henan 450008 China; 2 Infertility Department, Beijing University of Traditional Chinese Medicine Dongzhimen Hospital Luoyang Hospital, Luoyang, Henan 471000 China; 3 Engineering Research Center of Modern Preparation Technology of Traditional Chinese Medicine, Ministry of Education, Shanghai University of Traditional Chinese Medicine, Shanghai, 201203 China; 4 Department of Histology and Embryology, National Key Laboratory of Reproductive Medicine and Offspring Health, School of Basic Medical Sciences, Nanjing, Jiangsu 211166 China; 5 Department of Reproductive Medicine, Henan Provincial Hospital of Traditional Chinese Medicine, Zhengzhou, Henan 450000 China

**Keywords:** Acrorus tatarinowii, Sperm function, BCL2/Bax/Caspase3, Apoptosis

## Abstract

**Purpose:**

To investigate the therapeutic potential of Acrorus tatarinowii in oligoasthenozoospermia and its related mechanism through modulation of the BCL2/Bax/Caspase3 signaling pathway.

**Methods:**

Initially, using the TCMSP and Disgenet databases, active ingredients of Acrorus tatarinowii were identified and their target genes associated with sperm-related diseases were elucidated.Subsequently, an oligoasthenozoospermia mouse model was induced and treated with Acrorus tatarinowii. Serum hormone levels were assessed by ELISA, testicular histopathology by HE staining, and target gene expression by qPCR and Western blotting.

**Results:**

Acrorus tatarinowii treatment significantly upregulated BCL2 expression in the testes of oligoasthenozoospermic rat. This was accompanied by improved histopathological features in testicular tissues, reduced LH and FSH levels in serum.

**Conclusion:**

Acrorus tatarinowii exerts therapeutic effects in oligoasthenozoospermia by regulating the BCL2/Bax/Caspase3 pathway, maybe by inhibiting apoptosis, and promoting germ cell proliferation. These findings highlight its potential as a natural remedy for male infertility associated with sperm function disorders.

**Supplementary Information:**

The online version contains supplementary material available at 10.1186/s12610-024-00247-w.

## Résumé

**Objectif:**

Étudier le potentiel thérapeutique *d’Acrorus tatarinowii* dans l’oligoasthénozoospermie (OAT) et son mécanisme sous-jacent via la modulation de la voie de signalisation BCL2/Bax/Caspase3.

**Méthodes:**

les bases de données TCMSP et Disgenet, ont été utilisées pour définir les ingrédients actifs *d’Acrorus tatarinowii* ainsi que leurs gènes cibles pouvant être associés aux pathologies spermatiques. Un modèle rongeur OAT induit a ensuite été traité avec *Acrorus tatarinowii*. Les niveaux sériques hormonaux ont été évalués par ELISA, l’histopathologie testiculaire a été analysée par une coloration hématoxyline/éosine (HE), et l’expression des gènes cibles et des protéines correspondantes a été suivie par qPCR et Western blotting, respectivement.

**Résultats:**

Le traitement par *Acrorus tatarino*wii a significativement augmenté l’expression de BCL2 dans les testicules des animaux OAT. Cela s’est accompagné d’une amélioration des caractéristiques histopathologiques des tissus testiculaires et d’une réduction des taux sériques de LH et de FSH.

**Conclusion:**

*Acrorus tatarinowii* exerce des effets thérapeutiques bénéfiques sur l'oligoasthénozoospermie en régulant la voie BCL2/Bax/Caspase3, probablement via une inhibition de l'apoptose, et en favorisant la prolifération des cellules germinales. Ces résultats soulignent son potentiel en tant que remède naturel contre l'infertilité masculine associée à des troubles de la fonction spermatique.

## Introduction

Oligoasthenozoospermia, commonly known as weak sperm syndrome, refers to reduced sperm count and/or motility in males [1–4]. Globally, an estimated 10% to 15% of reproductive-age men experience some form of oligoasthenozoospermia, with varying prevalence across regions and populations [5–7]. Factors contributing to this condition include genetics, environmental exposures, lifestyle choices, and systemic diseases [8–10]. Symptoms typically include low sperm count, abnormal sperm morphology, and impaired motility, collectively impacting fertility [11, 12]. Treatment modalities encompass pharmacotherapy, surgical interventions, lifestyle adjustments, and assisted reproductive technologies tailored to individual etiology and clinical context [13–15]. Thus, a comprehensive understanding of the pathogenic mechanisms of oligoasthenozoospermia and treatment strategies is pivotal for enhancing male reproductive health.

Traditional Chinese Medicine (TCM) shows considerable therapeutic potential in managing oligoasthenozoospermia, presenting promising avenues for both research and clinical treatment [16–18]. Recent studies underscore the TCM efficacy in enhancing sperm quality and fertility parameters through multifaceted approaches [19, 20]. Investigations have focused on elucidating the molecular mechanisms underlying TCM interventions, which often involve modulation of oxidative stress, inflammation, hormone regulation, and apoptotic pathways crucial to spermatogenesis [21]. By targeting pivotal molecules such as TNF, MAPKs, and BCL2 family proteins, TCM aims to rejuvenate sperm function and promote male reproductive health [4, 22, 23].Prior research has highlighted Iris tenuifolia as another TCM effective in addressing sperm-related disorders, owing to its bioactive compounds such as 8-isopentenylkaempferol, kaempferol, eugenol, and cycloartenol [24, 25]. These compounds have demonstrated significant bioavailability and drug-likeness, thereby potentially influencing key molecular targets as*s*ociated with sperm function.

Herein, we aim to explore which components of Iris tenuifolia influence the progression of oligoasthenozoospermia and the signaling pathways they may regulate.This research may provide proposing novel therapeutic strategies for managing oligoasthenozoospermia through targeted molecular pathways.

## Materials and methods

### Pharmacology network analysis

Active Compound Identification of Acorus tatarinowii: A total of 105 chemical constituents of Acorus tatarinowii were retrieved from the TCMSP database (https://old.tcmsp-e.com/tcmsp.php). Using the criteria of oral bioavailability (OB) ≥ 30% and drug-likeness (DL) ≥ 0.18, four active compounds—8-isopentenylkaempferol, kaempferol, eugenol, and cycloartenol—were identified. Details of these compounds are presented in Table 1. Target genes for the active compounds were obtained using the TCMSP database, standardized via the UniProt database to remove duplicates, resulting in 80 unique targets.

**Table 1:** The primer sequece of target gene

Target name	F(5’-3’)	R(5’-3’)
TNF-α	CAGGCGGTGCCTATGTCTC	CGATCACCCCGAAGTTCAGTAG
MAPK14	TGACCCTTATGACCAGTCCTTT	GTCAGGCTCTTCCACTCATCTAT
GSPT1	CCAAGCCTTTCGTCCCCAA	TCTTGAGAGGACTCTACAGGTTC
KDR	CAAACCTCAATGTGTCTCTTTGC	AGAGTAAAGCCTATCTCGCTGT
HOMX1	AGGTACACATCCAAGCCGAGA	CATCACCAGCTTAAAGCCTTCT
GSTM1	ATACTGGGATACTGGAACGTCC	AGTCAGGGTTGTAACAGAGCAT
ESR1	CCCGCCTTCTACAGGTCTAAT	CTTTCTCGTTACTGCTGGACAG
CYP1B1	CCACCAGCCTTAGTGCAGAC	GGCCAGGACGGAGAAGAGT
CYP1A1	CAATGAGTTTGGGGAGGTTACTG	CCCTTCTCAAATGTCCTGTAGTG
BCL2	GCTACCGTCGTGACTTCGC	CCCCACCGAACTCAAAGAAGG
GAPDH	AGGTCGGTGTGAACGGATTTG	GGGGTCGTTGATGGCAACA

Target Identification for Sperm-Related Disorders: Using the DisGeNET database (https://www.disgenet.org/), five categories related to sperm-related disorders were searched: “Abnormality of the endocrine system,” “Deficiency of testosterone biosynthesis,” “Asthenozoospermia,” “Teratozoospermia,” and “Male infertility.” After filtering for redundancy, 558 unique disease-related targets were identified. The “VennDiagram” package in R was employed to find intersecting targets between active compounds and disease-related targets, producing a Venn diagram.

Drug-Compound-Target Network Construction: Using Cytoscape v3.8.0, a drug-compound-target network was constructed based on the active compounds and intersecting targets associated with sperm-related disorders. Notably, no intersecting targets were found for eugenol and cycloartenol with sperm-related diseases.

Protein–Protein Interaction (PPI) Network and Key Target Screening: The intersecting targets were uploaded to the STRING database (https://cn.string-db.org/) to conduct PPI network analysis, with settings specified as “Homo sapiens” and a minimum interaction score of 0.4. Irrelevant proteins were hidden, and network visualization and modifications were performed using Cytoscape. The top 10 key targets, ranked by “Count” values, were identified and visualized in a bar chart using R.

GO and KEGG Pathway Enrichment Analysis: GO and KEGG enrichment analyses of intersecting targets were conducted using the “enrichplot” and “ggplot2” packages in R. GO enrichment was categorized into biological process (BP), cellular component (CC), and molecular function (MF), with the top 10 terms in each category visualized in bar and bubble charts. A total of 944 GO terms were identified (864 BP, 12 CC, and 68 MF), while KEGG analysis identified 62 signaling pathways.

### Experimental animal

SPF-grade male SD rats weighing 200–220 g were purchased from Nanjing Keke Biological Company. Rats were randomly divided into control group, oligoasthenozoospermia group, L-carnitine group, Acrorus tataruibowii group, and Acrorus tataruibowii + Navitoclax group, with 10 rats per group. Oligoasthenozoospermia was induced in rats by intraperitoneal injection of cyclophosphamide (125 mg/kg) for 3 consecutive days. After model establishment, control group and model group received intragastric administration of equal volumes of physiological saline for 4 weeks.

### qPCR

After euthanizing the rats, tissues were collected and homogenized to obtain tissue homogenates. The homogenates were lysed in 1 mL Trizol (Invitrogen) lysis reagent. Subsequently, 200 μL chloroform was added, followed by vigorous shaking and incubation at room temperature for 3 min. Samples were then centrifuged at 12,000 rpm at 4 °C for 15 min. Isopropanol was added to the supernatant, followed by a 15-min incubation at room temperature and centrifugation. The RNA pellet was dissolved in DEPC-treated water and subjected to reverse transcription to generate cDNA products. Gene expression levels were analyzed using ABI QuantStudio 5, and Ct values were obtained for different samples. Gene expression data were analyzed using the 2^-ΔΔCT method. Gene primers are listed in Table 1.

### Western blot

The tissue homogenates were lysed in 1 × SDS buffer to extract protein samples. The protein samples were separated by SDS-PAGE gel electrophoresis. Subsequently, proteins were transferred onto membranes and incubated with primary antibodies against TNF-α (CST), MAPK14 (CST), KDR (CST), HMOX1 (CST), GSTP1 (CST), GSTM1 (Proteintech), ESR1 (Proteintech), CYP1B1 (Proteintech), CYP1A1 (Proteintech), and BCL2 (Abcam). After overnight incubation with primary antibodies, membranes were incubated with secondary antibodies. Chemiluminescent detection and imaging were performed using the Tanon 5200 fully automated chemiluminescence imaging system.

### ELISA

Approximately 2 mL of rat blood was collected and lysed with red blood cell lysis buffer to release cells. Subsequently, assays for T (Beyotime Biptech), E2 (Beyotime Biptech), LH (Sangon Biotech), and FSH (Sangon Biotech) were performed using respective ELISA kits. Initially, 100 μL of blood sample was added to antibody-coated plates and incubated at 37 °C for 1 h, followed by washing the plates six times. HRP-conjugated antibodies were then added and incubated at 37 °C for 30 min. TMB substrate solution was added for color development, and the reaction was terminated with stop solution. Absorbance values of different sample groups were measured using a dual-wavelength spectrophotometer.

### HE staining

The testicular tissues of rats were initially fixed in 4% paraformaldehyde for tissue preservation. Following fixation, tissues underwent dehydration in ethanol. Dehydrated samples were then infiltrated and hardened in melted paraffin wax. Subsequently, tissue samples embedded in paraffin were sectioned into thin slices (typically 5–10 µm thick) using a microtome. Deparaffinized sections were sequentially immersed in Harris hematoxylin staining solution for nuclear staining, typically for 15 min. After rinsing, sections were then immersed in eosin Y staining solution for cytoplasmic staining, also for 15 min. Prepared slides were observed under a microscope using 10X and 20X magnification objectives to examine the stained tissue structures and cellular morphology.

### Data analysis

Data analysis was uesd the Graphpad 8.0. Comparisons between two groups were used the t-test. *P* < 0.05 indicated that the difference was significant.

## Results

### The active ingredients prediction of Acrorus tatarinowii and its potential targets in regulating Sperm Function

**Fig. 1:**
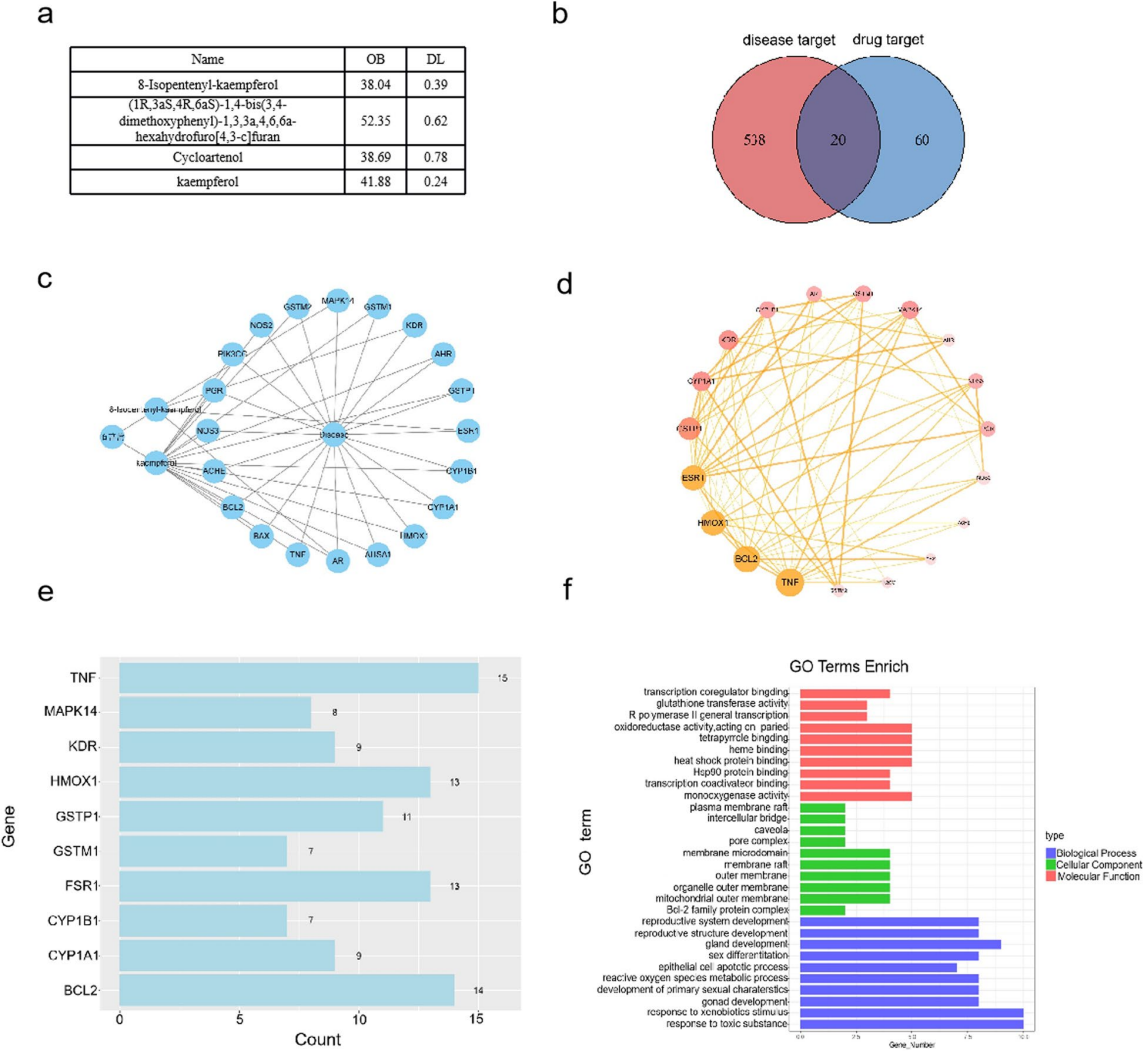
The active ingredients prediction of Acrorus tatarinowii and its potential targets in regulating Sperm Function. **a** Compound information table. **b** Venn diagram of active ingredients and their relevance to sperm-related diseases. **c**, **d**, **e** Differential gene analysis.f. GO pathway enrichment analysis

Initially, according to the TCMSP database (https://old.tcmsp-e.com/tcmsp.php), we retrieved 105 chemical components of Iris tenuifolia, filtering them with criteria of OB (oral bioavailability) ≥ 30% and DL (drug-likeness) ≥ 0.18. This yielded four effective ingredients: 8-isopentenylkaempferol, kaempferol, eugenol, and cycloartenol. Specific information on these compounds is detailed in Table 1a. Subsequently, using the Disgenet database (https://www.disgenet.org/), we searched for target genes associated with five sperm-related diseases: “Abnormality of the endocrine system,” “Deficiency of testosterone biosynthesis,” “Asthenozoospermia finding,” “Teratozoospermia,” and “Male infertility.” This search yielded 558 non-redundant target genes. Using the R programming language and the “VennDiagram” package, we identified intersection targets between the active ingredients and sperm diseases, visualizing them with a Venn diagram (Fig. 1b).

Next, we visualized the Iris tenuifolia active ingredients and their intersection targets with diseases, constructing a drug-active ingredient-intersecting target network by using Cytoscape v3.8.0 software,. Key targets were identified by ranking the top 10 based on Count values using R, including TNF, MAPK14, KDR, HMOX1, GSTP1, GSTM1, ESR1, CYP1B1, CYP1A1, and BCL2 (Fig. 1c-e). Finally, using the R packages “enrichplot” and “ggplot2,” we conducted GO and KEGG enrichment analyses on the intersection targets. GO enrichment analysis included biological processes (BP), cellular components (CC), and molecular functions (MF), revealing that Iris tenuifolia affects sperm functions primarily involved in regulating oxidative stress and sperm cell differentiation (Fig. 1f).

### Therapeutic effects of Acrorus tatarinowii and its effects on the expression of weak sperm-related targets

**Fig. 2:**
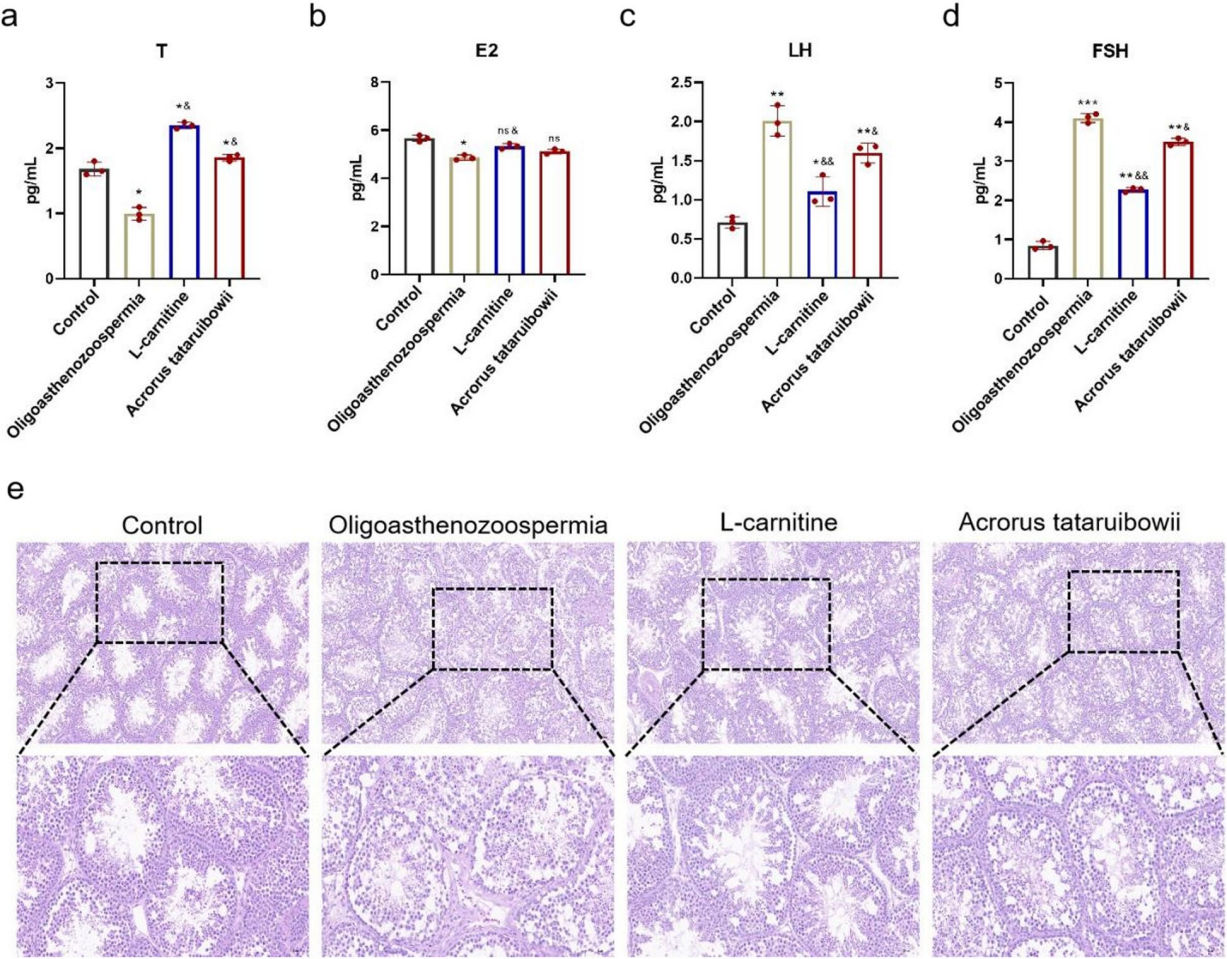
Pathological analysis of Acorus tatarinowii treatment on oligoasthenozoospermia in rats. **a**-**d** ELISA detection of changes in serum T, E2, LH, and FSH levels in rats. **e** HE staining to assess testicular tissue damage in rats. *n* = 3,vs.control,*P < 0.05,**P < 0.01, vs.Oligoasthenozoospermia,^&^*P* < 0.05,ns,no significance

**Fig. 3:**
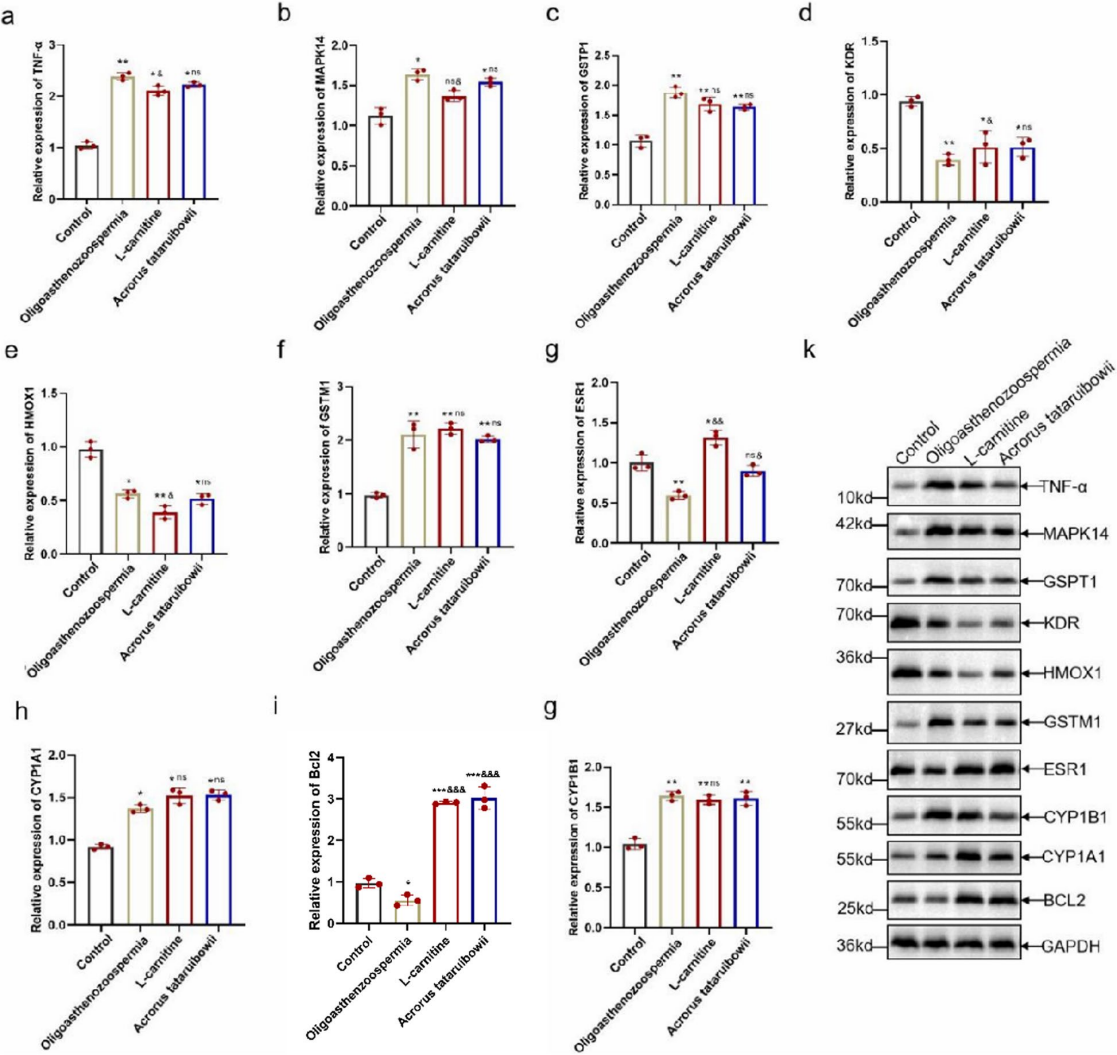
Acrorus tatarinowii controls the BCL2-related pathway in the oligoasthenozoospermia rat. **a**-**j** qPCR analysis of mRNA expression of TNF-α, MAPK14, GSPT1, KDR, HMOX1, GSTM1, ESR1, CYP1B1, CYP1A1, and BCL2 in rat testicular tissues. K Western blot detection of protein expression of each gene. *n* = 3,vs.control,*P < 0.05,**P < 0.01,***P < 0.001 vs.Oligoasthenozoospermia,^&^*P* < 0.05,^&&^*P* < 0.01, ^&&&^*P* < 0.001, ns, no significance

Rats were intraperitoneally injected with cyclophosphamide (125 mg/kg) for three consecutive days to induce oligoasthenozoospermia, with a control group receiving physiological saline treatment. Subsequently, rats were treated with Acrorus tatarinowii. After one week of treatment, rat were euthanized. ELISA was used to measure serum levels of LH and FSH, revealing significant reductions in LH and FSH levels in the oligoasthenozoospermic rat. Following Acrorus tatarinowii treatment, levels of LH and FSH in serum increased(Fig. 2a-d). HE staining was used to assess pathological changes in the testicular tissues of oligoasthenozoospermic rat(Fig. 2e). The model group exhibited significant pathological changes in seminiferous tubules and interstitial tissues, including degenerative necrosis and disordered arrangement of various levels of spermatogenic cells, vacuolization of cells, interstitial edema, and congestion. In contrast, testicular tissue damage in the Acrorus tatarinowii treatment group was improved. To further validate potential target genes predicted by network pharmacology, protein expression of TNF, MAPK14, KDR, HMOX1, GSTP1, GSTM1, ESR1, CYP1B1, CYP1A1, and BCL2 in the testes of oligoasthenozoospermic rat was examined using qPCR (Fig. 3a-j)and Western blotting(Fig. 3k,S1). The results showed significant upregulation of BCL2 expression after Acrorus tatarinowii treatment.In addition, after Acrorus tatarinowii treatment, the protein expression levels of TNF-α, MAPK14, GSPT1, KDR, CYP1A1, CYP1B1, and HOMX1 were significantly reduced, while the protein expression of ESR1 was significantly increased (Fig. 3k, S1).

### Acrorus tatarinowii controls the BCL2-related pathway in the oligoasthenozoospermia rat

**Fig. 4:**
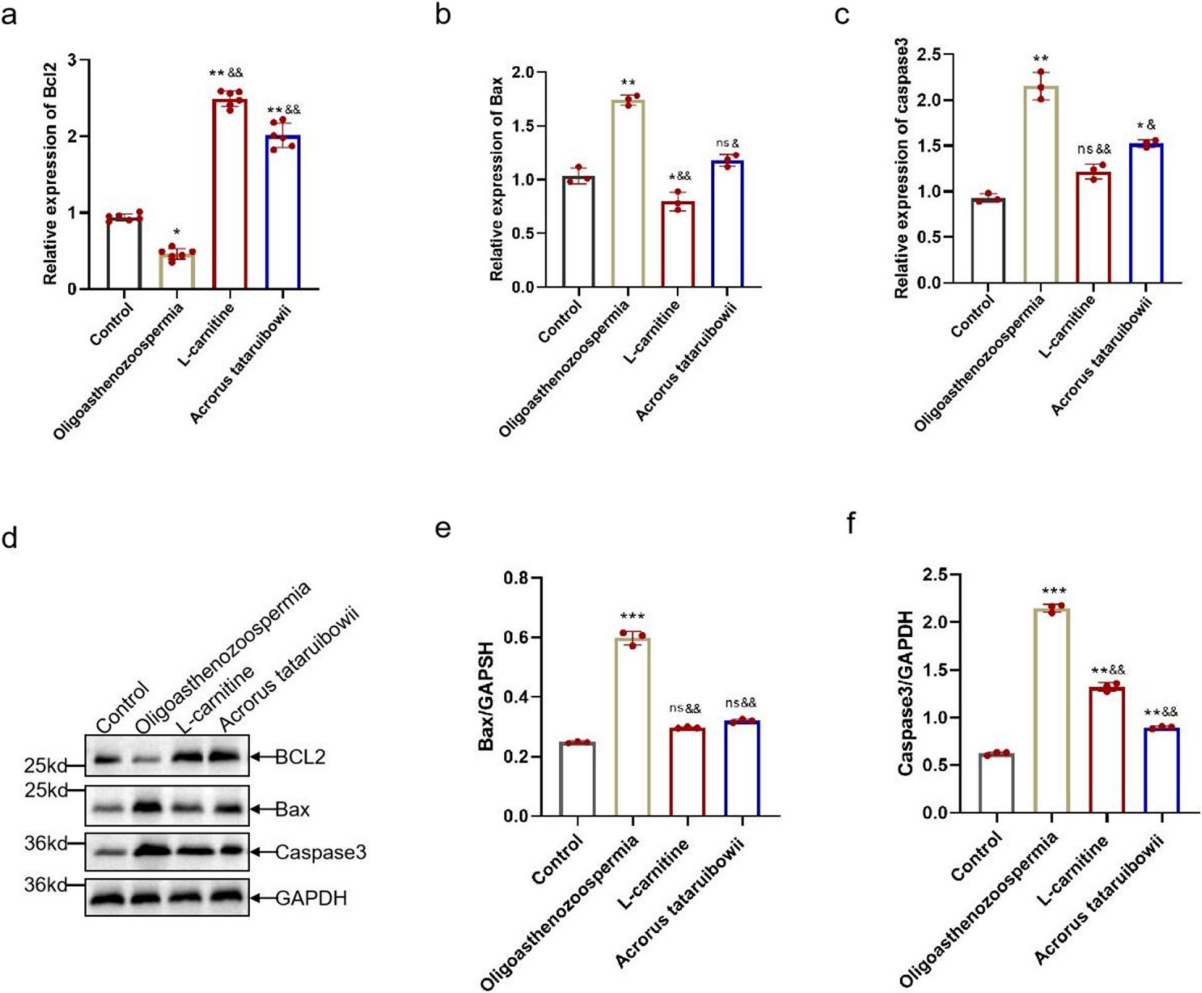
Analysis of BCL2-related signaling protein expression in rats treated with Acrorus tatarinowii. **a**-**c** qPCR detection of mRNA expression of BCL2, Bax, and caspase3. **d**-**e** Western blot detection of protein expression of BCL2, Bax, and caspase3. *n* = 3,vs.control,*P < 0.05,**P < 0.01,***P < 0.001 vs.Oligoasthenozoospermia,^&^*P* < 0.05,^&&^*P* < 0.01, ns, no significance

Next, we aimed to further confirm whether Acorus tatarinowii induces changes in BCL2-associated signaling molecules in alleviating oligoasthenozoospermia. BCL2 is a classic anti-apoptotic protein [26]. Here, we assessed the expression of two critical downstream molecules, Bax and Caspase3 [27]. It has shown that BCL2 negatively regulates the expression of Bax and Caspase3 to inhibit cell apoptosis. Following administration of Acorus tatarinowii, we examined the mRNA and protein expression of BCL2 and Caspase3 in rat testicular tissue(Fig. 3a-c). Results indicated a significant decrease in BCL2 and Caspase3 protein expression compared to the model group(Fig. 3d-f). Hence,we speculated that Acorus tatarinowii on oligoasthenozoospermia may influence the vitality of germ cells in oligoasthenozoospermia rat (Fig. 4).

### Navitoclax impaires the inhibition of Acrorus tatarinowii on the Bcl2/Bax/Caspase3

**Fig. 5:**
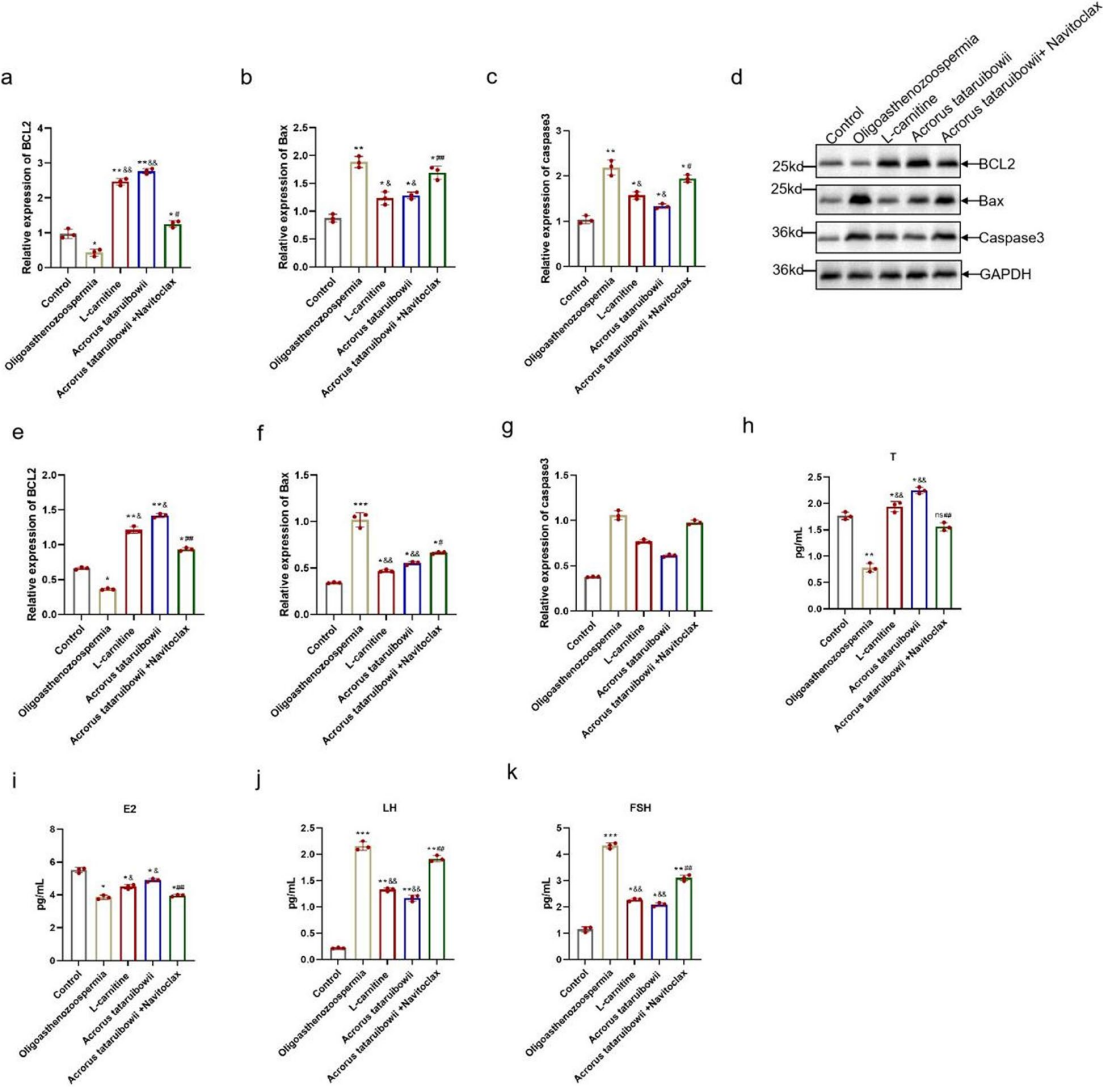
Navitoclax impaires the inhibition of Acrorus tatarinowii on the Bcl2/Bax/Caspase3. **a**-**c** mRNA expression of BCL2, Bax, and caspase3. **d**-**g** Protein expression of BCL2, Bax, and caspase3. **h**–**k** ELISA detection of T, E2, LH, and FSH levels.*n* = 3,vs.control,*P < 0.05,**P < 0.01,***P < 0.001 vs.Oligoasthenozoospermia,^&^*P* < 0.05,^&&^*P* < 0.01, ^&&&^*P* < 0.001, vs.Acrorus tataruibowii, #*P* < 0.05, ##*P* < 0.01,###*P* < 0.001, ns, no significance

To confirm that Acorus tatarinowii treats oligoasthenozoospermia by modulating the BCL2/Bax/Caspase3 signaling pathway, we employed the BCL2 inhibitor Navitoclax in combination with Acorus tatarinowii in rats. It showed that compared to the Acorus tatarinowii group, BCL2 expression significantly decreased, while Bax and Caspase3 expression significantly increased in the Navitoclax co-treatment group(Fig. 5a-g). This indicates that Navitoclax attenuated the regulatory effects of Acorus tatarinowii on BCL2. Furthermore, levels of T, E2, LH, and FSH were measured. In the Navitoclax co-treatment group, T and E2 levels significantly decreased, whereas LH and FSH levels significantly increased(Fig. 5h-k). Additionally, HE staining revealed more severe testicular tissue damage in the Navitoclax-treated group. Thus, in oligoasthenozoospermia, Acorus tatarinowii alleviates asthenozoospermia symptoms effectively through modulation of the BCL2/Bax/Caspase3 pathway.

## Discussion

Male infertility, characterized by impaired sperm function and production, poses a significant global health challengehenozoospermia, a common form of male infertility, is marked by reduced sperm count and motility, complicating natural conception [28]. Traditie medicine (TCM) has long been utilized for male infertility, with herbal remedies playing a notable role in alleviating symptoms associated with low sperm count and motility [29]. This study integrated computational bioinformatics and in vivo experimental approaches to explore the therapeutic mechanisms of Acorus tatarinowii in treating oligoasthenozoospermia.

Initially, bioinformatics analysis identified active compounds of Iris tenuifolia, specifically 8-isopentenylkaempferol, kaempferol, eugenol, and cycloartenol, as well as their potential targets relevant to sperm health. To connect these targets to sperm-related diseases, we utilized a multi-step approach.Previous studies have employed RNA sequencing and transcriptomic profiling to reveal regulatory pathways and therapeutic mechanisms in conditions like varicocele, a common cause of male infertility. One study examining the therapeutic effects of Morinda officinalis polysaccharide on varicocele utilized sequencing to pinpoint specific long non-coding RNAs and mRNAs as regulatory factors, underscoring how TCM components can influence gene expression linked to male reproductive health [28].Herein, using the TCMSP and Disgenet databases, we identified 558 non-redundant target genes associated with sperm dysfunction, which we then narrowed down by focusing on intersecting genes with known effects on reproductive processes. This filtering identified key sperm-related targets, including TNF, MAPK14, KDR, HMOX1, GSTP1, ESR1, CYP1B1, and BCL2. Our analysis underscored that these proteins are integral to pathways linked to sperm motility, oxidative stress response, and apoptotic regulation. Through Cytoscape analysis, we visualized the complex network interactions between the compounds and their sperm-related targets, offering insights into the specific molecular mechanisms by which Acorus tatarinowii exerts its therapeutic effects.

In vivo experiments using a cyclophosphamide-induced mouse model further validated the therapeutic efficacy of Acorus tatarinowii. Specifically, Acorus tatarinowii treatment normalized serum levels of luteinizing hormone (LH) and follicle-stimulating hormone (FSH), two hormones critical for spermatogenesis. Elevated levels of these hormones counteracted cyclophosphamide-induced hormonal disruptions, as observed in oligoasthenozoospermia. Histopathologicalevealed significant improvements in testicular tissue architecture, such as reduced degenerative changes in seminiferous tubules and interstitial cells. Importantly, the upregulation of BCL2 suggests a mechanism that protects germ cells from apoptosis, thereby promoting their survival and potential proliferation.

The role of the BCL2/Bax/Caspase3 pathway in mediating Acorus tatarinowii’s therapeutic effects is particularly noteworthy. Acorus tatarinowii treatmd BCL2 expression, effectively suppressing apoptosis within testicular tissues. However, when co-administered with the BCL2 inhibitor Navitoclax, the anti-apoptotic effects were countered, resulting in significant increases in Bax and Caspase3 expression levels, as well as elevated apoptosis. Navitoclax’s modulation of these key proteins supports the hypothesis that Acorus tatarinowii’s beneficial effects rely on the BCL2 pathway, highlighting its potential as a targeted therapy for oligoasthenozoospermia by directly influencing apoptotic regulation.This finding is reinforced by studies where pathway-focused bioinformatic analyses were applied to uncover cell division cycle genes as well as apoptotic factors in varicocele, highlighting potential therapeutic targets for male infertility [29].

Given that reactive oxygen species (ROS) are major mediators of male infertility, it is essential to consider how Acorus tatarinowii may influence ROS-related pathways. Elevated ROS levels have been closely associated with oxidative stress, a condition that damages sperm DNA and impairs motility. By downregulating oxidative stress-related proteins such as HMOX1 and MAPK14, Acorus tatarinowii may indirectly reduce ROS levels, thereby mitigating oxidative stress in testicular tissues. This aligns with recent findings suggesting that oxidative stress regulation is a key target in managing male infertility, and supports our observation that Acorus tatarinowii enhances sperm quality and viability by stabilizing ROS-related pathways [30, 31].

In conclusion, this study provides mechanistic insights into the therapeutic potential of Acorus tatarinowii in treating oligoasthenozoospermia. Through modulating the BCL2/Bax/Caspase3 pathway and regulating oxidative stress-related targets, Acorus tatarinowii demonstrates significant promise in improving sperm quality and viability. Future research should focus on clinical trials to assess its safety and efficacy in human subjects with male infertility. Additionally, exploring synergistic effects with conventional therapies and optimizing treatment regimens could enhance therapeutic outcomes and broaden the application of TCM in reproductive medicine.

## Supplementary Information

Supplementary Material 1: S1 TNF-α, MAPK14, GSPT1, KDR, HMOX1, GSTM1, ESR1, CYP1B1, CYP1A1, BCL2 protein expression quantitative analysis. *n* = 3,vs.control,*P < 0.05,**P < 0.01,***P < 0.001 vs.Oligoasthenozoospermia,^&^*P* < 0.05,^&&^*P* < 0.01, ns, no significance.

## Data Availability

The datasets used and/or analyzed during the current study are available from the corresponding author on reasonable request.
