# Supplementary figures and images for: The protective effects of active ingredients from acrorus tatarinowii on sperm and their molecular mechanisms
Source: Basic Clin Androl. 2025 Jan 20;35:2. doi: 10.1186/s12610-024-00247-w (PMC11744876; doi:10.1186/s12610-024-00247-w)

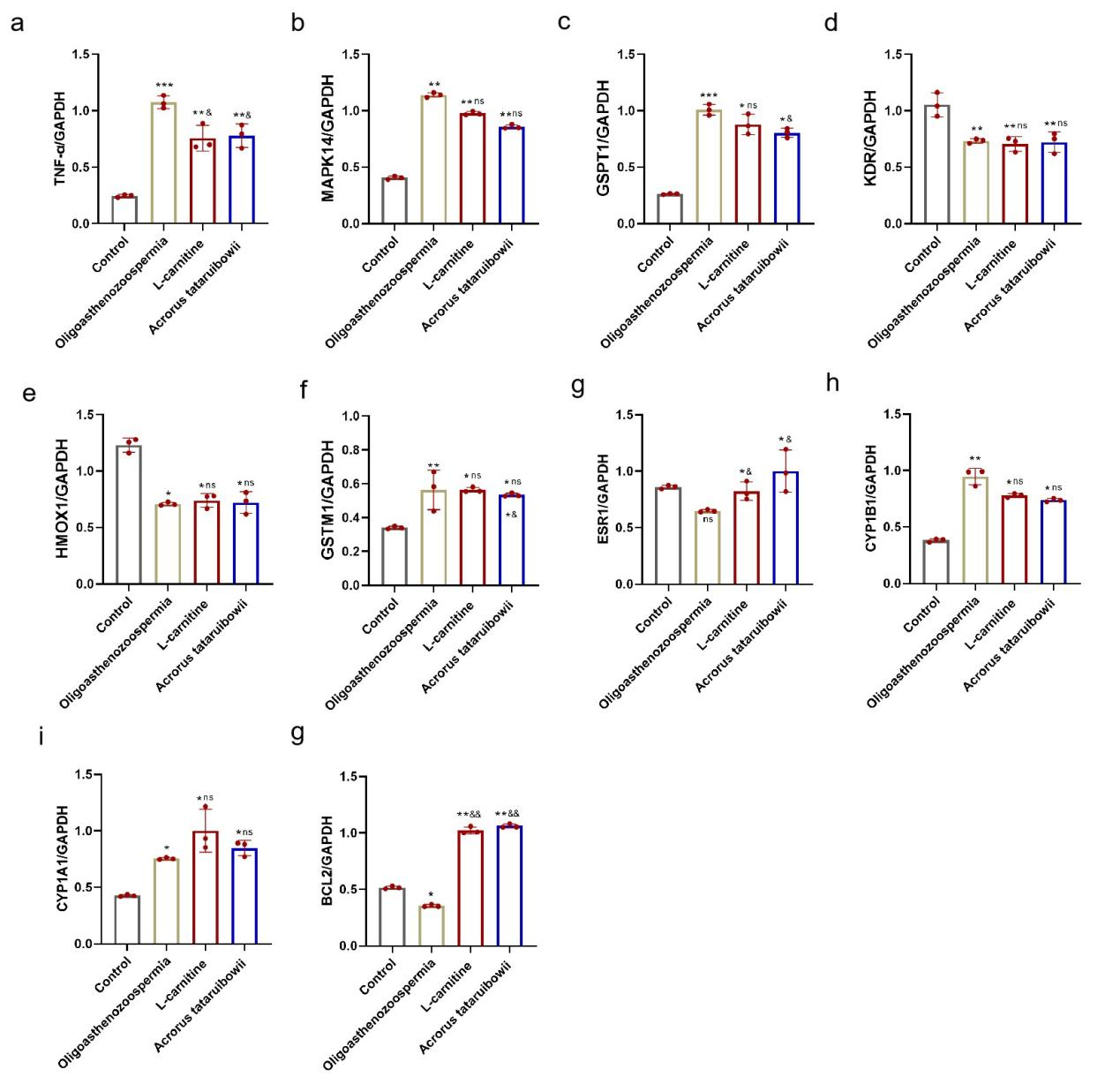

Supplement: Supplementary file 1 — Supplementary Material 1: S1 TNF-α, MAPK14, GSPT1, KDR, HMOX1, GSTM1, ESR1, CYP1B1, CYP1A1, BCL2 protein expression quantitative analysis. n = 3,vs.control,*P < 0.05,**P < 0.01,***P < 0.001 vs.Oligoasthenozoospermia,&P < 0.05,&&P < 0.01, ns, no significance. [file 12610_2024_247_MOESM1_ESM.png]
